# Microarray analysis points to LMNB1 and JUN as potential target genes for predicting metastasis promotion by etoposide in colorectal cancer

**DOI:** 10.1038/s41598-024-72674-8

**Published:** 2024-10-10

**Authors:** Jiafei Liu, Hongjie Yang, Peng Li, Yuanda Zhou, Zhichun Zhang, Qingsheng Zeng, Xipeng Zhang, Yi Sun

**Affiliations:** 1 Department of Colorectal Surgery, Tianjin Union Medical Center, Tianjin, People’s Republic of China; 2 Tianjin Institute of Coloproctology, Tianjin, People’s Republic of China; 3 The Institute of Translational Medicine, Tianjin Union Medical Center of Nankai University, Tianjin, People’s Republic of China

**Keywords:** Chemotherapy, Etoposide, Colorectal cancer, LMNB1, JUN, Cancer, Cell biology, Biomarkers, Diseases, Gastroenterology, Oncology

## Abstract

Etoposide is a second-line chemotherapy agent widely used for metastatic colorectal cancer. However, we discovered that etoposide treatment induced greater motility potential in four colorectal cancer cell lines. Therefore, we used microarrays to test the mRNA of these cancer cell lines to investigate the mechanisms of etoposide promoting colorectal cancer metastasis. Differentially expressed genes (DEGs) were identified by comparing the gene expression profiles in samples from etoposide-treated cells and untreated cells in all four colorectal cancer cell lines. Next, these genes went through the Gene Set Enrichment Analysis (GSEA), Gene Ontology (GO) and the Kyoto Encyclopedia of Genes and Genomes (KEGG) Pathway analysis. Among the top 10 genes including the upregulated and downregulated, eight genes had close interaction according to the STRING database: FAS, HMMR, JUN, LMNB1, MLL3, PLK2, STAG1 and TBL1X. After etoposide treatment, the cell cycle, metabolism-related and senescence signaling pathways in the colorectal cancer cell lines were significantly downregulated, whereas necroptosis and oncogene pathways were significantly upregulated. We suggest that the differentially expressed genes LMNB1 and JUN are potential targets for predicting colorectal cancer metastasis. These results provide clinical guidance in chemotherapy, and offer direction for further research in the mechanism of colorectal cancer metastasis.

## Introduction

A second-line chemotherapeutic agent, etoposide is used for chemotherapy in advanced and metastasis colorectal cancer patients^1,2^. Etoposide causes the breaks of double-stranded DNA and topoisomerase by stabilizing the DNA break complex^3^. It activates endonuclease in the body and acts on DNA through its metabolites^4^. In a preliminary experiment, we discovered that a 12 h treatment with 20 μM etoposide promoted metastasis in colorectal cancer cells. This observation manifests the phenomenon of chemotherapy-induced metastasis widely reported in recent research^5,6^. Although metastatic diseases account for about 90% of cancer-related deaths, future research on cancer metastasis is still needed to form a unifying conceptual framework^7^.

Previous research has shown that metastasis can be facilitated by cancer metabolism upregulation^8^, which in turn can be stimulated by many oncogenes such as mTOR kinase^9^. Via scrutinizing the mRNA expressions of four colorectal cancer cell lines, we demonstrate that metabolism-related pathways are upregulated and oncogenes are activated after etoposide treatment; hence, intracellular oncogene activation and metabolic upregulation work together to induce cancer metastasis.

All four colorectal cancer cell lines used in this study are KRAS-mutated^10^. About 35–45% colorectal cancers patients have the mutated KRAS gene^11^, the high-frequency mutations being substitutions in codons 12&13. KRAS-mutated genetic testing has become the key decision for classifying colorectal patients. For example, in cetuximab p.G13D-mutated patients have better overall survival than KRAS-mutated patients, since p.G13D-mutated cancer cells sensitivity in cetuximab^12^. In our study, the cell lines HCT116, LoVo and DLD1 are p.G13D-mutated in KRAS, and SW480 is p.G12V-mutated^10^.

Microarrays is a simple and fast detection method for detecting gene expression which are used in screening for DEGs^13^. A significant amount of data produced by microarray analysis is available in public databases. Previous studies concerning colorectal cancer gene expression profiling have identified hundreds of DEGs^14–17^. In our study, we first identified DEGs by comparing the gene expression data in samples from etoposide-treated cells and untreated cells. Next, these genes went through the GSVA, GO and the KEGG PATHWAY analysis. Protein–Protein Interaction (PPI) network was performed. Interrelation between the gene pathways was also examined for these genes. These bioinformatic analyses pinpointed two genes central to the PPI network as novel drug targets: JUN and LMNB1. Immune cells with overexpressed JUN have strengthened cell motility, migration and invasion. Overexpress JUN engineering immune cells rendered them resistant to exhaustion^18^. Increased activity of fatty acid pathways leads to JUN activation, as saturated fatty acids activate Jun N-terminal kinase^19^. Deficiency nuclear lamin B1 (LMNB1) induced defects associating with malignancy function in human hematopoietic stem cell. The loss of LMNB1 altered genome organization and instability since nonfully-functional DNA damage repair^20^, reducing LMNB1 increased senescence^21^.

## Materials and methods

### Cell cultures

The human colorectal cancer cell lines HCT116, LoVo, SW480 and DLD1 were purchased from the ATCC (Manassas, VA, USA). HCT116, LoVo and SW480 cells were cultured in RPMI-1640 cell culture medium (Gibco, Thermo Fisher Scientific, Shanghai, China) appending with 10% fetal bovine serum (FBS; Gibco, Life Technologies, New Zealand) and 1% penicillin–streptomycin^22^ (Gibco, Life Technologies, USA). DLD1 cells were cultured in DMEM high sugar medium (HyClone, Thermo Fisher Scientific, Shanghai, China) appeding with 10% fetal bovine serum (FBS; Gibco, Life Technologies, New Zealand) and 1% penicillin–streptomycin^22^ (Gibco, Life Technologies, USA). Both cell lines were cultured at 37 °C in a humidified atmosphere containing 5% CO_2_.

### Wound-healing assay

The migratory ability of HCT116, LoVo, SW480 and DLD1 cells was evaluated by wound-healing assays. The cells were incubated in a 24-well plate, drawing at the back of the plate with three lines. Next, the cells were cultured overnight. Straight scratches were made on the back, while the plate was gently washed twice with PBS removing excess cells. Finally, cells were cultured either in 20 μM etoposide or in regular medium, both kept at 37 °C with 5% CO_2_ observed after 12 h.

### Cell migration and invasion assays

Cell migration assay was performed in 24-well CIM plates (BD Biosciences, CA, USA)^22^. Briefly, 10,000 to 30,000 cells per well were seeded in serum-free medium with indicated drugs in the upper compartment of the CIM plates. Serum-complemented medium was added to the lower compartment of the chamber^22^. After a 12 h incubation, fixing with cold methanol and staining with crystal violet, counting the four random microscopic fields. Cell invasion assay was performed in 24-well CIM plates coated with matrigel. Other steps were identical to those of cell migration assay^22^. Etoposide was provided by HengRui Medicine (Jiangsu, China).

### RNA extraction and quantitative real-time PCR

Total RNA was extracted from cell pellets using Trizol reagent (Invitrogen) following the specification. RNA samples with an OD260/OD280 ratio between 1.9 and 2.0 were used for the following cDNA synthesis using High capacity RNA to cDNA kits (Promega, Madison, WI, USA)^22^. Quantitative PCR was performed using SYBR Green master mix (Applied Biosystems) with the housekeeping gene GAPDH as the internal control. The relative expressions of LMNB1 and JUN were calculated using the comparative Ct method. The primers of LMNB1, JUN and GAPDH were as follows:

- LMNB1 forward, 5′-AAGCATGAAACGCGCTTGG-3′,
- LMNB1 reverse, 5′-AGTTTGGCATGGTAAGTCTGC-3′;
- JUN forward, 5′-ACT AAG CTT GCC GCC ACC ATG ACT GCA AAG ATG GAA ACG AC-3′,
- JUN reverse, 5′-GAG GGA TCC TCA AAA CGT TTG CAA CTG CTG-3′;
- GAPDH forward, 5′-GGAGCGAGATCCCTCCAAAAT-3′,
- GAPDH reverse, 5′-GGCTGTTGTCATACTTCTCATGG-3′.

### Proliferation assay

Primary cells were seeded in 96-well plates and treated as needed. Cell viability was measured 24\48\72\96 h after treatment cessation using the Cell-Counting Kit 8 (CCK-8) (40203ES80) from shanghai Yesen biotechnology. Plates were incubated for 1.5 h at 37 °C and absorbance was measured at 450 nm using the Infinite 200 Tecan i-control plate reader machine. The CCK-8 assay was performed in at least 3 replicates for each experimental condition^23^.

### Apoptosis staining assay

Cells were seeded in a 96-well plates and treated as needed. Cells apoptosis was tested using TUNEL apoptosis detection Kit (C1091) from shanghai Beyotime Biotechnology. After biotin labeling and subsequent DAB color development, apoptotic cells can be displayed in ordinary optical microscope (Olympus Corporation, Tokyo, Japan).

### Microarray data

Gene expression data was obtained using the Affymetrix PrimeView human gene expression array from 8 samples, among which 4 were treated with etoposide and 4 were not treated. The limma package (version 3.48.3) in R (version 4.0.1) was used to identify the DEGs between 4 etoposide-treated and 4 untreated colorectal cancer cell samples. We regarded genes with fold change ≥ 1.2 or ≤  − 1.2 and *p* < 0.05 as DEGs. The ggplot2 package (version 3.3.5) in R (version 4.0.1) was used to draw heatmaps for the top 20 significantly changed genes (top 10 upregulated and downregulated genes). All these data are available as supplementary materials.

### GO and KEGG pathway enrichment analyses

GO and KEGG pathway enrichment analysis of DEGs had been done in R (version 4.0.1). GO enrichment analysis was including biological process (BP), cellular component (CC), and molecular function (MF). *P* < 0.05 and gene counts of > 5 were considered to indicate a statistically significant difference. In addition, we performed GSVA on etoposide-treated and untreated samples using the GSVA package in R (1.40.1), with “c2.cp.kegg.v7.4.symbols.gmt” and “c5.all.v7.4.entrez.gmt” as basic gene sets.

### The gene set enrichment analysis (GSEA)

We performed GSEA on etoposide-treated and untreated cancer cells samples using the clusterprofiler package (version 4.0.5) in R, defining pathways significative *p* < 0.05.

### Integration of the PPI network

The STRING database (version 11.5) was used for analyzing PPI network and the interrelationship in pathways in order to evaluate the PPI network among DEGs.

## Results

### DEG analysis for etoposide-treated colorectal cancer cells

**Fig. 1:**
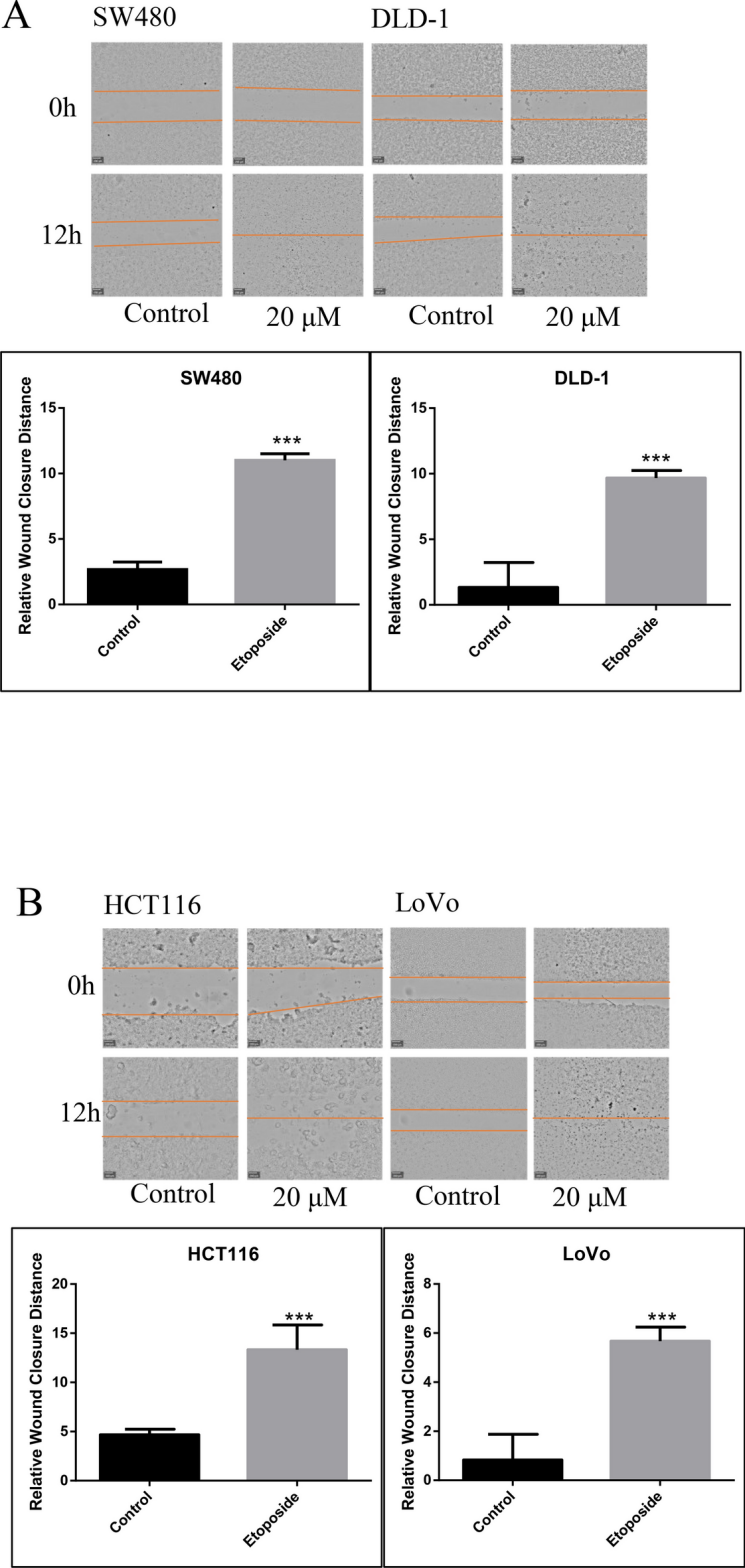
(**A**) Wound-healing assay in SW480 and DLD-1 cells before and after etoposide treatment at 0 h and 12 h (100 ×). Column diagram showing the comparison between wound-healing abilities in SW480 and DLD-1 cells before and after etoposide treatment (***P < 0.001). (**B**) Wound-healing assay in HCT116 and LoVo cells before and after etoposide treatment at 0 h and 12 h (100 ×). Column diagram showing the comparison between wound-healing abilities in HCT116 and LoVo cells before and after etoposide treatment (***P < 0.001).

**Fig. 2:**
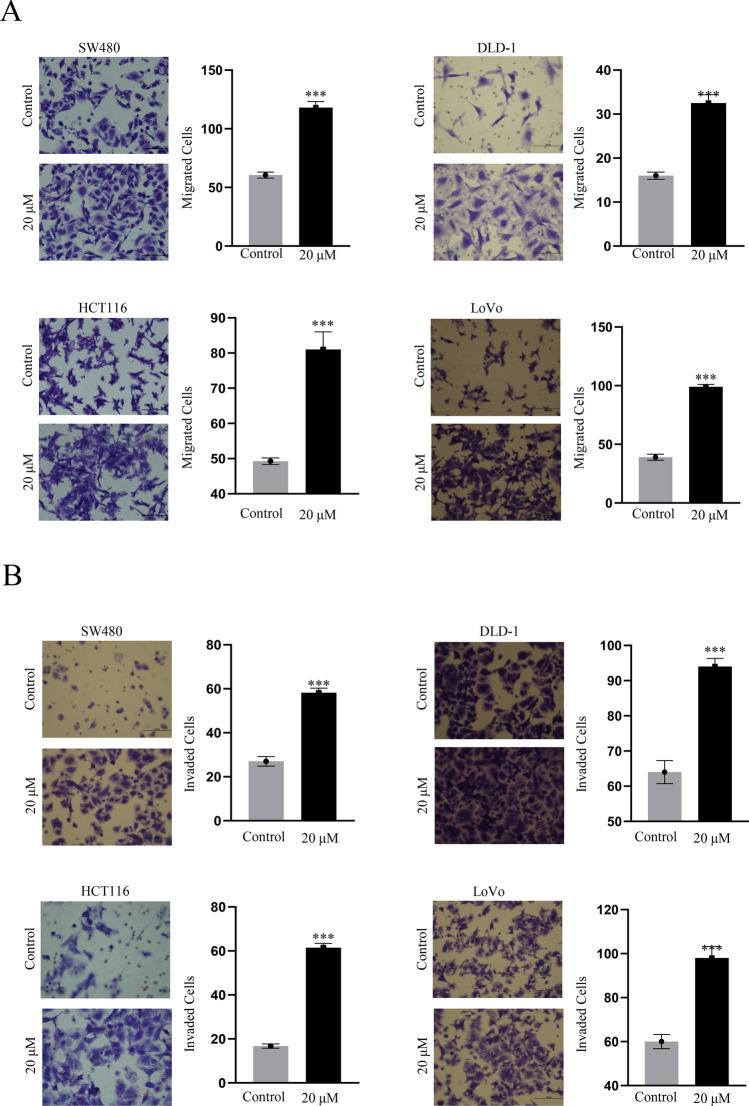
(**A**) Etoposide promotes the migration of HCT116, LoVo, SW480 and DLD1 cells. Cells were subjected to transwell chamber assays for 12 h in the presence of indicated concentrations of agents. (**B**) Etoposide promotes the invasion of HCT116, LoVo, SW480 and DLD1 cells. Cells were subjected to transwell chamber assays with Matrigel for 12 h in the presence of indicated concentrations of agents.

Etoposide promoted the motility of colorectal cancer cells. The statistical data of the wound-healing assay showed a significant enhance in the migratory ability of HCT116, LoVo, SW480 and DLD1 cell lines after a 12 h etoposide treatment in contrast to control groups (Fig. 1A,B). Although 20 μM etoposide inhibited cell proliferation (Supplementary Fig. 6A) and induced apoptosis (Supplementary Fig. 6B) in these cell lines. Cell migration and invasion results confirmed this phenomenon (Fig. 2A,B). The four cancer cell lines were selectively chosen according to our previous publication^22^, which demonstrated that different cancer cell lines have disparate responses to chemotherapeutic drugs. All four cell lines are KRAS-mutated (Supplementary Table 1). We identified DEGs by comparing the gene expression profiles of samples from etoposide-treated cell lines and untreated cell lines. To identify the DEGs, we performed DEGs analysis using the limma package (version 3.48.3) in R (version 4.0.1), and identified 6924 genes that were either up- or downregulated (Supplementary Table 2).

**Fig. 3:**
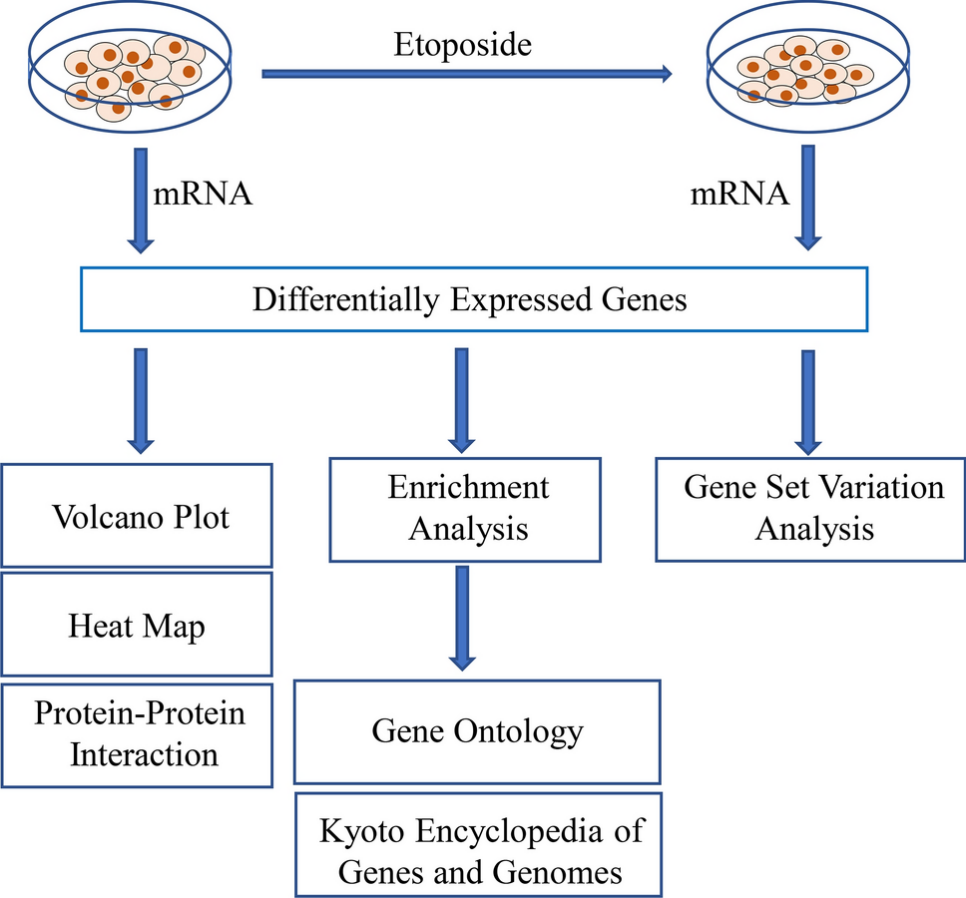
Flow chart of the study.

**Fig. 4:**
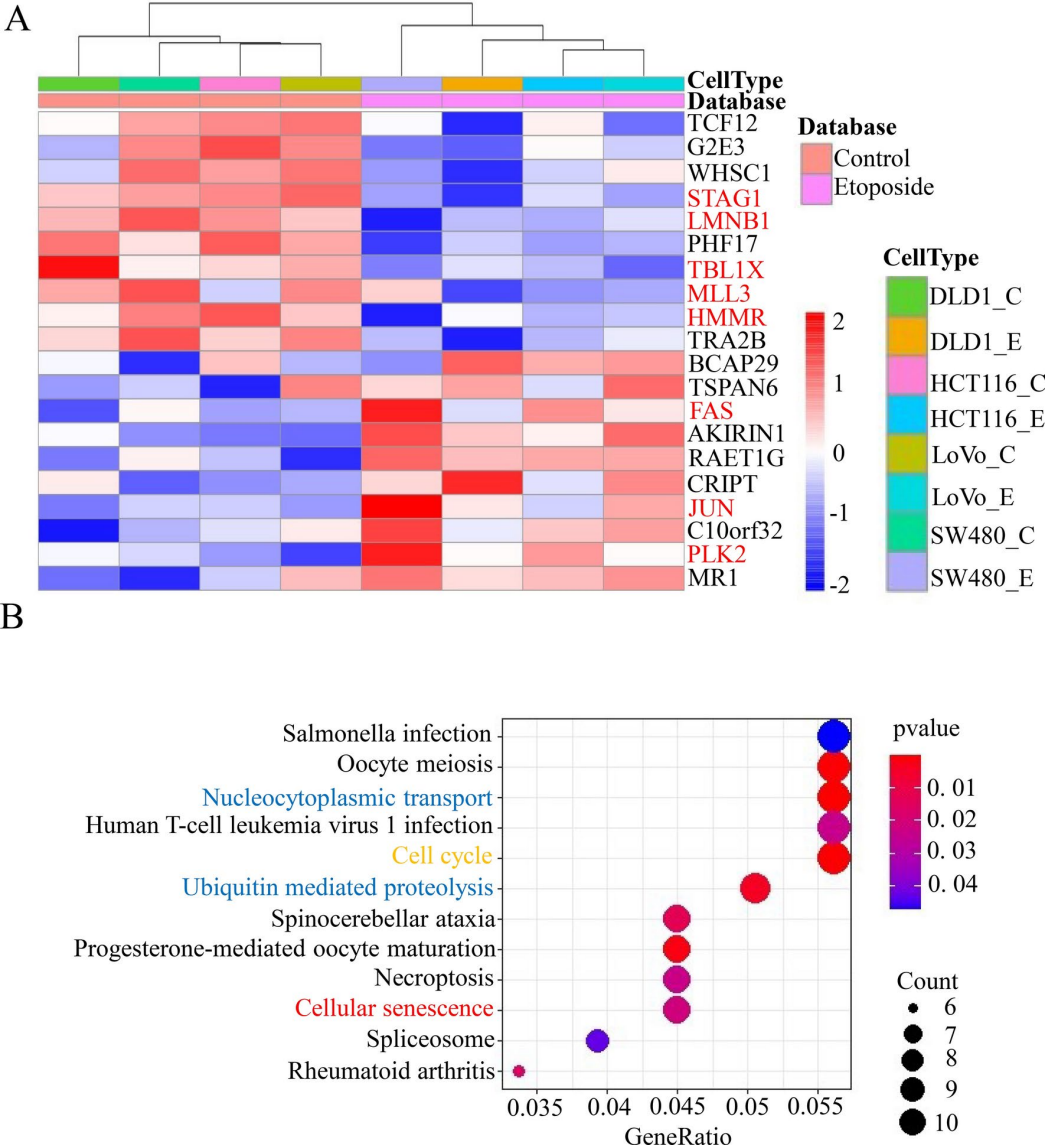
(**A**) Heatmap of the top 20 DEGs (top 10 up-and top 10 downregulated genes). Red ones represent upregulation and blue represent downregulation. (**B**) Significantly KEGG enriched terms obtained in the twelve gene sets.

Integrative analysis was conducted on cell DEGs after a 12 h 20 μM etoposide treatment (Fig. 3). Between the 4 etoposide-treated and 4 untreated colorectal cancer cell samples, the expression levels of 6924 genes were up-or downregulated, among which 272 were significantly downregulated and 166 were significantly upregulated (Supplementary Fig. 1A). In Supplementary Fig. 1A, we also labeled the 8 genes selected via PPI analysis (see section “GSEA and PPI reveal the effect of etoposide on colorectal cancer cells”). Figure 3 shows a heatmap of the top 20 genes, with the 8 genes selected via PPI marked in red (Fig. 4A).

### Enrichment analysis of DEGs

Using the KEGG enrichment analysis database, 12 DEG enriching pathways matched our screening criteria of count > 5 and *p*-value < 0.05 (Fig. 4B), among which nucleocytoplasmic transport and ubiquitin mediated proteolysis were metabolism-related signaling pathways (labeled in mazarine). The 12 signaling pathways can be classified into four types: (1) intracellular invocation of cellular biological components, such as hsa03013; (2) cell cycle (labeled in yellow, Fig. 4B), such as hsa04110; (3) ubiquitin mediated proteolysis, ubiquinone and other terpenoid-quinone biosynthesis, necroptosis and autophagy, such as hsa04114, hsa04914 and hsa04120; (4) cellular senescence (labeled in red, Fig. 4B), such as hsa04218 and hsa04217. Types (1)–(3) are metabolism-related, while type (4) comprises senescence signaling pathways.

Therefore, after etoposide treatment, the cell cycle and metabolic related pathways of the colorectal cancer cell lines were significantly downregulated. Chemotherapeutic drug-induced cell cycle decline had an overlaying advantage over changes in other cellular pathways. Under the combined action of superposition advantages and overlay advantages, metabolism-related signaling pathways assume dominant roles in cancer metastasis.

To better understand the significantly enriched pathways, we performed enrichment analysis separately in the 166 up-and 272 downregulated gene sets. In all the 166 upregulated gene sets, we identified one meaningfully enriched pathway (count > 5, *p*-value < 0.05), that was hsa04217: necroptosis (count = 6, *p*-value = 0.00203). This means that in the etoposide-treated colorectal cancer cell lines, the necroptosis pathway was significantly upregulated, resulting in an overwhelming preponderance in DEG. In all the 272 downregulated gene sets, three vital pathways were enriched (count > 5, *p*-value < 0.05), which were hsa03013: nucleocytoplasmic transport, hsa04120: ubiquitin mediated proteolysis, hsa04110: cell cycle (Supplementary Fig. 2A). This demonstrated that the metabolism-related signaling pathways nucleocytoplasmic transport and ubiquitin mediated proteolysis might be vital to etoposide promoting metastasis.

### KEGG pathway analysis of DEGs

The Kyoto Encyclopedia of Genes and Genomes (KEGG) gene enrichment heat map demonstrated that 34 up-and 37 downregulated gene sets were considered as significantly changed differentially expressed gene sets subject to the criteria of fold change ≥ 1.5 or ≤  − 1.5 and *p*-value < 0.05. In comparison with the control group, the 34 upregulated gene sets of the etoposide-treated group could be classified into 3 groups: (1) oncogene signaling pathway (Supplementary Fig. 2B), such as mTOR signaling pathway, TGF-β signaling pathway, VEGF signaling pathway, WNT signaling pathway; (2) immunity (Supplementary Fig. 2C), such as intestinal immune network, FC epsilon RI signaling pathway, chemokine signaling pathway; (3) metabolism-related signaling pathways, such as biological macromolecule metabolism, amino acid metabolism signaling pathways, nucleotide metabolism signaling pathways (Supplementary Fig. 2D). In comparison with the control group, the 37 downregulated gene sets of the etoposide-treated group could be classified into 4 groups: (1) tumor suppressor genes signaling pathway, such as the p53 signaling pathway; (2) cell tight junction, such as adhesion molecules and junction; (3) cell cycle; (4) cell transcription and metabolism (Supplementary Fig. 3A).

### GO term enrichment analysis of DEGs

BP analysis (Supplementary Table 3) showed that there were 14 statistically significant (count > 5, *p*-value < 0.05) upregulated DEGs: nine were enriched in metabolism-related signaling pathways (labeled in mazarine, Supplementary Fig. 3B), three were enriched in oncogene signaling pathways (labeled in crimson, Supplementary Fig. 3B), and two were enriched in immunity pathways (labeled in loden, Supplementary Fig. 3B). The upregulation of the 14 pathways above may be an important cause of etoposide promoting tumor metastasis. This aligns with the KEGG-GSVA database enrichment analysis results. There were 17 statistically significant (count > 5, *p*-value < 0.05) downregulated DEGs (Supplementary Table 3), which were mainly enriched in four categories: (1) downregulation of cell proliferation and division, such as regulation of mitotic cell cycle phase transition, cell cycle checkpoint (labeled in yellow, Supplementary Fig. 4A); (2) downregulated tumor suppressor gene, such as signal transduction by p53 class mediator (labeled in crimson, Supplementary Fig. 4A); (3) inhibition of DNA damage repair, such as signal transduction in response to DNA damage, DNA damage checkpoint and response, p53 class mediator signal transduction, telomere maintenance (labeled in brown, Supplementary Fig. 4A); (4) cytoskeleton, such as microtubule organizing center organization, regulation of microtubule cytoskeleton organization (labeled in cerise, Supplementary Fig. 3A).

Cellular Component analysis (Supplementary Table 4) showed that there were 2 statistically significant (count > 5, *p*-value < 0.05) upregulated DEGs (Supplementary Table 4). They were metabolism-related signaling pathway vacuolar membrane (labeled in mazarine, Supplementary Fig. 4B) and autophagy related pathway autophagosome (labeled in violet, Supplementary Fig. 4B). There were 7 statistically significant (count > 5, *p*-value < 0.05) downregulated DEGs (Supplementary Table 4). They were mainly enriched in three categories: (1) cell cycle (labeled in yellow, Supplementary Fig. 5A); (2) cytoskeleton (labeled in crimson, Supplementary Fig. 5A); (3) transcription (labeled in orange, Supplementary Fig. 5A).

Molecular Function analysis (Supplementary Table 5) showed that there were 2 statistically significant (count > 5, *p*-value < 0.05) upregulated DEGs (Supplementary Table 5). They were metabolism-related signaling pathways GTP binding and GTPase activity (labeled in mazarine, Supplementary Fig. 5B). There were 6 statistically significant (count > 5, *p*-value < 0.05) downregulated DEGs (Supplementary Table 5) mainly enriched in two classifications: (1) cytoskeleton (labeled in crimson, Supplementary Fig. 5C); (2) transcription (labeled in orange, Supplementary Fig. 5C).

### GSEA and PPI reveal the effect of etoposide on colorectal cancer cells

**Fig. 5:**
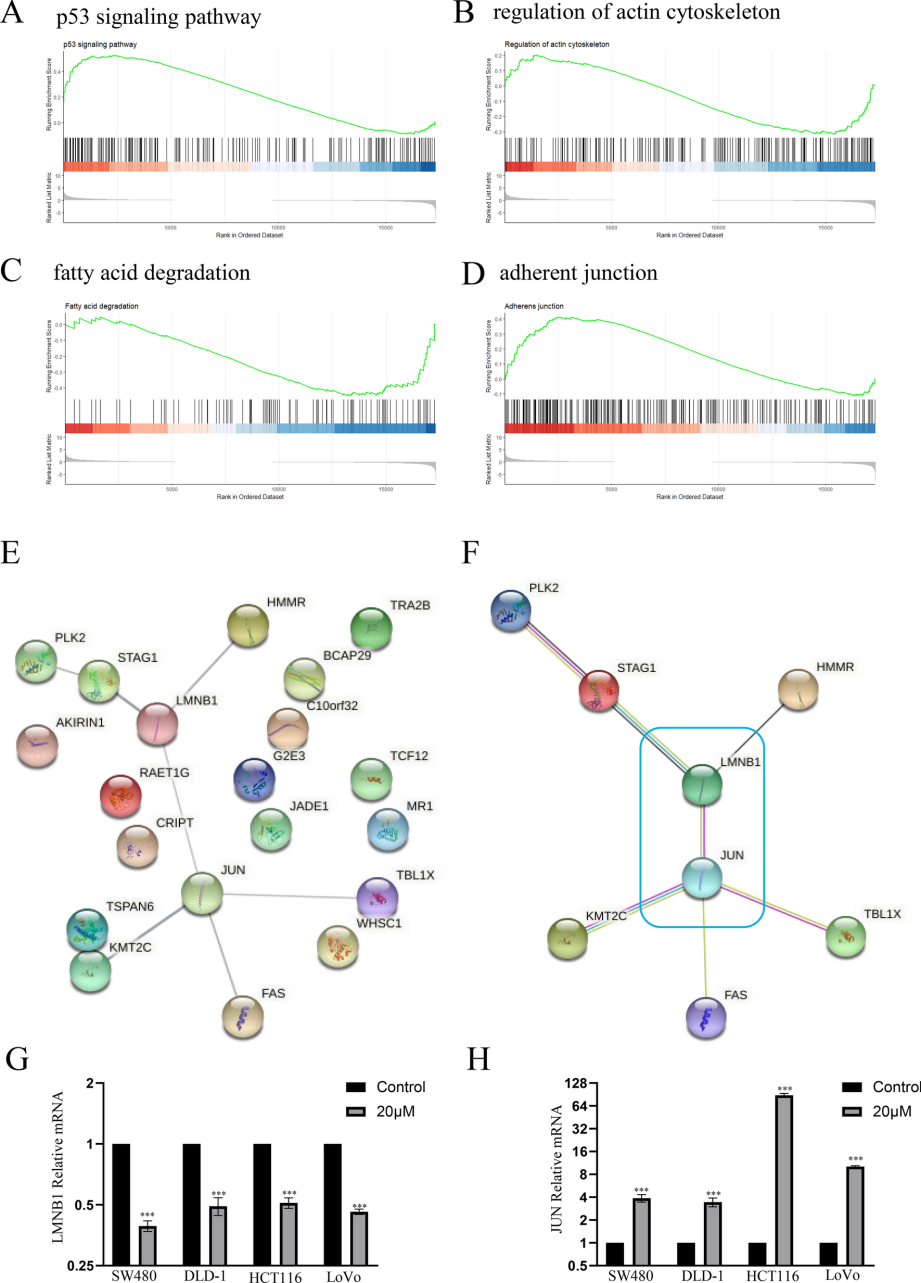
(**A**) GSEA showing the enrichment of p53 signaling pathway in colorectal cancer cells after etoposide stimulation. (**B**) GSEA showing the enrichment of regulation of actin cytoskeleton in colorectal cancer cells after etoposide stimulation. (**C**) GSEA showing the enrichment of fatty acid degradation in colorectal cancer cells after etoposide stimulation. (**D**) GSEA showing the enrichment of adherens junction in colorectal cancer cells after etoposide stimulation. (**E**) The PPI network of 20 top regulated DEGs obtained using STRING. (**F**) The PPI network of PLK2, STAG1, LMNB1, HMMR, KMT2C, JUN, FAS and TBL1X obtained using STRING. (**G**) qRT-PCR analysis of LMNB1 mRNA after treatment with etoposide (20 μM). (**H**) qRT-PCR analysis of JUN mRNA after treatment with etoposide (20 μM).

Etoposide stimulation mainly altered four gene sets: (1) p53 signaling pathway, (2) regulation of actin cytoskeleton, (3) fatty acid degradation, (4) adherens junction (Fig. 5A–D). PPI network analysis on the top 10 upregulated and downregulated DEGs using the STRING database (Fig. 5E) shows close interactions between 8 genes: PLK2, STAG1, LMNB1, HMMR, KMT2C, JUN, FAS and TBL1X. Moreover, LMNB1 and JUN were central to the PPI network (Fig. 5F). Correlated with increased JUN mRNA expression and decreased LMNB1 mRNA expression shown in the microarray, the level of LMNB1 mRNA was downregulated in cell lines after etoposide treatment, while the level of JUN mRNA was upregulated (Fig. 5G,H).

## Discussion

Cancer metastasis is the main cause of high mortality^24^. Although chemotherapy have side effects, the clinical effect is well reflected on survival demonstrated in several tumors^25–27^. However, chemotherapy-induced metastasis has also come to light. Previously, we found that chemotherapy topoisomerase inhibitors could promote the ROS-elevated expression and secretion of CXCL1, activating JAK2-STAT1 signaling pathway and inactivating PTP1B, thereby promoting the migration and invasion of cancer cells^22^. The paclitaxel through increasing pre-metastatic microenvironment density enhance tumor metastasis in mice^28^. In breast cancer paclitaxel drives metastasis in mice, regulated by the non-cancer host cells and stress-inducible gene Atf3^6^. Paclitaxel also help tumor creating a metastasis favorable environment by over secreted annexin-6 from tumor-derived exosomes^29^. The side effects of chemotherapy in promoting metastasis in tumor treatment are gradually being understood.

**Fig. 6:**
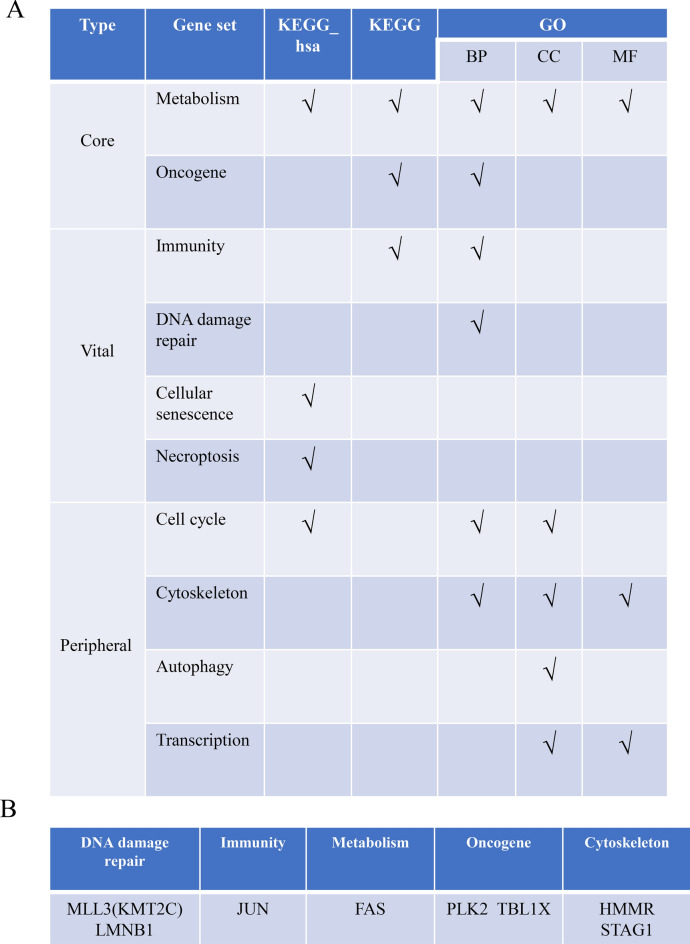
(**A**) Summary of enrichment pathway analysis. (**B**) Summary of the PPI network of PLK2, STAG1, LMNB1, HMMR, KMT2C, JUN, FAS and TBL1X obtained using STRING.

To summarize, from the KEGG and GO enrichment analyses, we found out that the DEGs were mainly concentrated in the metabolism-related pathways and oncogene pathways, as well as in four categories including immunity, DNA damage repair, cellular senescence and necroptosis pathways (Fig. 6A). The GSVA bioinformation analysis further affirmed these pathways. Among the 8 genes selected from the PPI network, FAS, JUN and PLK2 were upregulated, HMMR, MLL3, LMNB1, STAG1 and TBL1X were downregulated. While JUN and LMNB1 were central to the PPI network, the six peripheral genes were also closely related to the core pathways, vital pathways and peripheral pathways (Fig. 6B). (1) FAS: FAS is a fatty acid synthetase gene, as a key enzyme in the synthesis of fatty acids, has rich enzyme system functions, and it exists in different forms in human body, and it plays a great role in affecting the energy metabolism of organisms. In triple-negative breast cancer, the expression of fatty acid synthetase is positively correlated with Ki-67 proliferation index^30^. Qinxi Li et al. found that FAS-mediated apoptosis could support colorectal cancer survival^31^. In adipocytes FAS signal pathway promotes colorectal cancer lung metastasis and low-grade inflammation^32^. (2) PLK2: PLK2 is a serine threonine protein kinase which promotes tumor multiplication and inhibits apoptosis in colorectal cancer^33^. From 7 GEO datasets including overall survival data found that FOXD1 and PLK2 were identified as the independent risk prognostic factor. FOXD1 and PLK2 were detected through IHC staining in 131 CRC patients’ pathological section tissue. Patients with high expression of FOXD1 and PLK2 had poor disease-free survival and overall survival^34^. In CRC PLK2 might be an independent prognostic marker. In CRC PLK2 inhibits apoptosis and promotes tumor growth. Moreover, PLK2 through binding Fbxw7 to stabilizing Cyclin E. PLK2 represents an independent prognostic marker by targeting Fbxw7/Cyclin E^33^. (3) HMMR: HMMR is a hyaluronan mediated motility receptor gene crucial to centrosome functions and mitotic spindle integrity^35^. A level of HMMR outside of an appropriate range is not conducive to the stability of cell centrosomes. Thus, aggressive cancers have extreme expression value of HMMR both including abnormally high and low expression. For several types of cancers such as pancreatic islet tumor and breast cancer, poor patient survival rate is associated with low HMMR expressions^35^. Survival analysis revealed that HMMR was significantly associated with overall survival of CRC patients^36^. In GSE21510 data filtering the different expression genes between CRC tissues and noncancerous tissues, identifying AURKA, BUB1, DLGAP5 and HMMR. These genes were associated for regulating oocyte meiosis, mitotic cycle phase transition, finding their mechanisms in drug-sensitive therapeutic targets of CRC^37^. (4) MLL3: MLL3, also called KMT2C, is one of the SET1/MLL family members of histone H3K4 methyltransferases. MLL3 is an enhancer in monomethylating histone H3K4. MLL3 mutation forms were common in many kinds of tumors. MLL3 is beneficial to the repair of DNA damage^38^. In acute myeloid leukemia and colorectal cancer there is MLL3 gene germ line mutation from exome sequencing^39^. Here, the restoration of KMT2C/MLL3 reducing CRC cell growth through reinforcing H3K4me1 deposition at enhancers; however, in KMT2C/MLL3 deficient cells their H3K4me1 status affecting varied states. The non-functional KMT2C/MLL3 promote colorectal cancer development by dysregulation in transcriptional pathways^40^. MLL3 frameshift mutations in CRC cells and primary tumors are more common in cases of microsatellite instability. In addition, the CpG island-associated promoter of the MLL3 gene has no DNA methylation in CRC cells, and no DNA methylation in the primary tumor and normal colon, and this region has a highly homologous pseudogene (psiTPTE22), which is associated with age-related DNA methylation^41^. (5) STAG1: STAG1 enhances the proliferation of tumor cells. Simultaneously blocking STAG1 family member STAG2 reduces cell proliferation^42^. STAG1 is the encoding core subunits for cohesin genes^43^. STAG2, the most frequently mutated subunit in the binding protein complex, exhibits a strong synthetic lethal interaction with its parallel STAG1. Mechanistically, STAG1 loss disrupts sister chromatid cohesion in Stag2-mutated cells but not in wild-type cells, leading to mitotic mutations, defects in cell division and apoptosis. STAG1 inactivation inhibits the proliferation of STAG2 mutations but not in Ewing’s sarcoma and bladder cancer. In mutant bladder cancer models, restoring STAG2 expression mitigated dependence on STAG1. Therefore, STAG1 and STAG2 support sister chromatid cohesion to ensure cell survival. STAG1 represents the vulnerability of cancer cells that carry mutations in the major emerging tumor suppressor STAG2 in different cancer environments. Using synthetic lethal interactions to target recurrent cohesive protein mutations in cancer, for example by inhibiting STAG1, holds promise for the diffusing of selective treatments^42^. (6) TBL1X: Transducin β-like protein 1 (TBL1) is an exchange adaptor protein encoded by the TBL1X gene and is known as regulating WNT signaling pathway by binding to β-catenin and promoting its downstream transcriptional program^44^. TBL1X was shown abnormally expression in diverse cancers^45^. We showed TBL1X expression was amusing higher in metastatic nasopharyngeal carcinoma tissues compared to non-metastatic tissues and correlated with TNM stage and metastasis of nasopharyngeal carcinoma patients. In addition, nasopharyngeal carcinoma patients with high TBL1X expression had a poor prognosis. TBL1X interacted with TCF4 to trans-activate Flot2 expression. TBL1X promoted nasopharyngeal carcinoma cell migration and invasion through Flot2. Moreover, Flot2 upregulated c-myc increasing the expression of TBL1X, as TBL1X’s positively regulatory transcription factor. TBL1X could reestablish the functional changes of nasopharyngeal carcinoma cells by Flot2 alteration. TBL1X and Flot2 have the same direction of regulation in nasopharyngeal carcinoma. TBL1X and Flot2 high expression patients got poorer disease-free survival and overall survival compared with any single one high expression of them. To promote nasopharyngeal carcinoma metastasis, TBL1X and Flot2 positively regulate each other providing new potential targets for nasopharyngeal carcinoma^45^.

Since JUN and LMNB1 are in the center of the PPI network, we claim that they are of crucial importance. JUN facilitates the spreading of tumors by boosting the body’s immune system. Overexpression JUN CAR-T cells enhanced expansion potential and functional capacity while diminishing terminal differentiation. JUN mediates the dysfunctioning of exhausted natural human T cells. Overexpression JUN CAR-T cells rendered to exhaustion resistant^20^. Saturated fatty acids through membrane subdomains induce c-Src clustering, resulting in an activation of Jun N-terminal kinase^21^. A loss of nuclear lamin B1 (LMNB1) encoding on chromosome 5q induces defects in malignancy hematopoietic stem cell. LMNB1 deficiency alters genome organization and instability because DNA damage repair defection^46^. Reducing Lamin B1 increases senescence^22^. Etoposide activates senescence and induces downregulation of Lamin B1^47^.

It is possible that these molecules LMNB1 and JUN act alone as markers of early colorectal cancer metastasis, or in combination with each other, but this requires validation by clinicians in clinical therapies. The oncogene LMNB1 affects DNA damage repair and hence serves as an application value therapeutic target in future therapy. JUN is involved in the fatty acid metabolic pathway corresponding to the enriched GSVA pathways. JUN also participates in cancer immunology and stem cell formation. Coordination of immune system components improves the survival rate after tumor transplantation, while the formation of stem cells is crucial to tumor transplantation^7^. In our previous research, microarray analysis revealed an interesting phenomenon where the chemotherapeutic drug etoposide significantly reduced cell proliferation^25^. Moverover, the significant decline of cell proliferation-related signaling pathways caused by this mechanism of action may cover up some other signaling pathways related to metastasis promotion, making it difficult for us to explore the promotion of colorectal cancer metastasis by the chemotherapeutic drug etoposide. Currently, single-cell sequencing is a good solution to this problem, and we recommend that institutions with sufficient research funds use single-cell sequencing for analysis.

## Conclusions

In conclusion, our research demonstrated that the major mechanisms behind the promotion of colorectal cancer metastasis by etoposide are intracellular metabolism upregulation and oncogene activation. LMNB1 and JUN are potential target genes for predicting colorectal cancer metastasis. Our finding provides clinical guidance in chemotherapy, and offers a direction for further research in the mechanism of colorectal metastasis.

## Supplementary Information

Supplementary Information 1.

Supplementary Information 2.

Supplementary Information 3.

Supplementary Information 4.

Supplementary Information 5.

Supplementary Information 6.

## Data Availability

Data is provided within the supplementary information files.

## Abbreviations

BP: Biological process
CC: Cellular component
DEGs: Differentially expressed genes
GO: Gene ontology
GSEA: Gene set enrichment analysis
GSVA: Gene set variation analysis
KEGG: Kyoto encyclopedia of genes and genomes
LMNB1: Lamin B1
MF: Molecular function
PPI: Protein–protein interaction
