# Supplementary material for: Microarray analysis points to LMNB1 and JUN as potential target genes for predicting metastasis promotion by etoposide in colorectal cancer
Source: Sci Rep. 2024 Oct 10;14:23661. doi: 10.1038/s41598-024-72674-8 (PMC11467296; doi:10.1038/s41598-024-72674-8)
Supplement: Supplementary file 6 — Supplementary Information 6. [file 41598_2024_72674_MOESM6_ESM.pdf]

Supplementary Figure 1

A

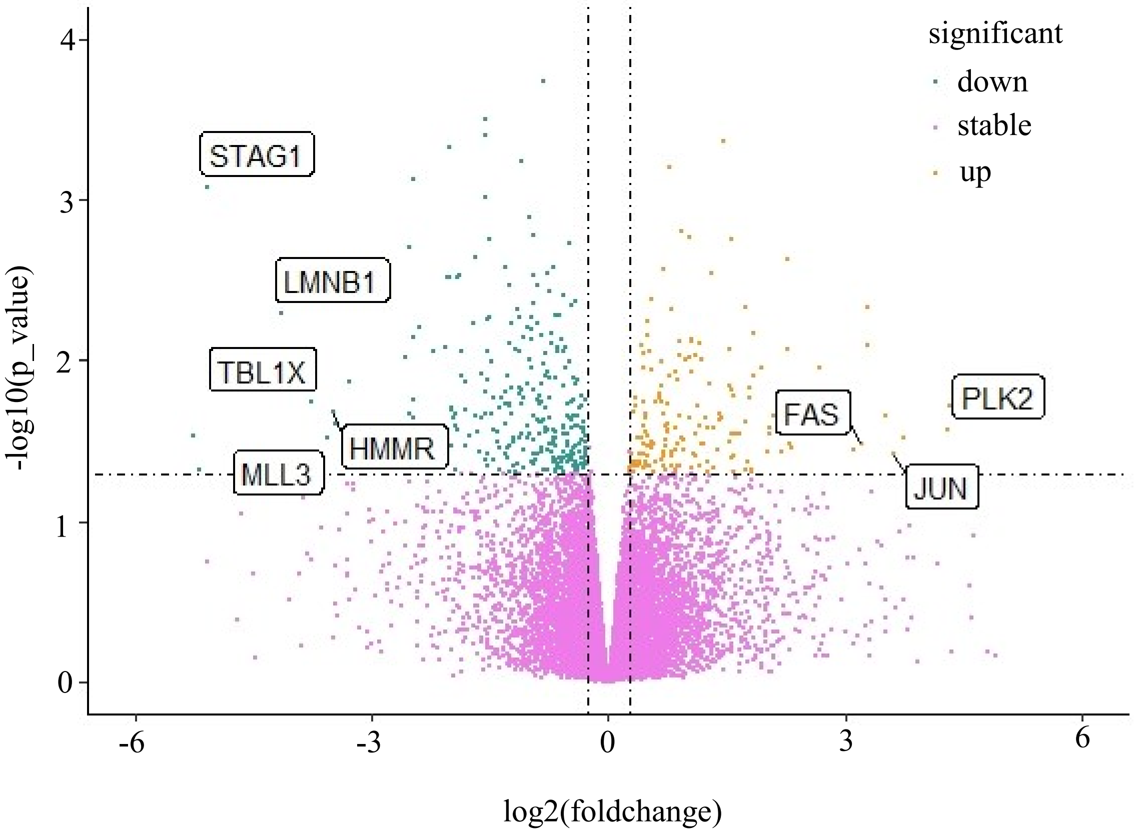

Supplementary Figure 2

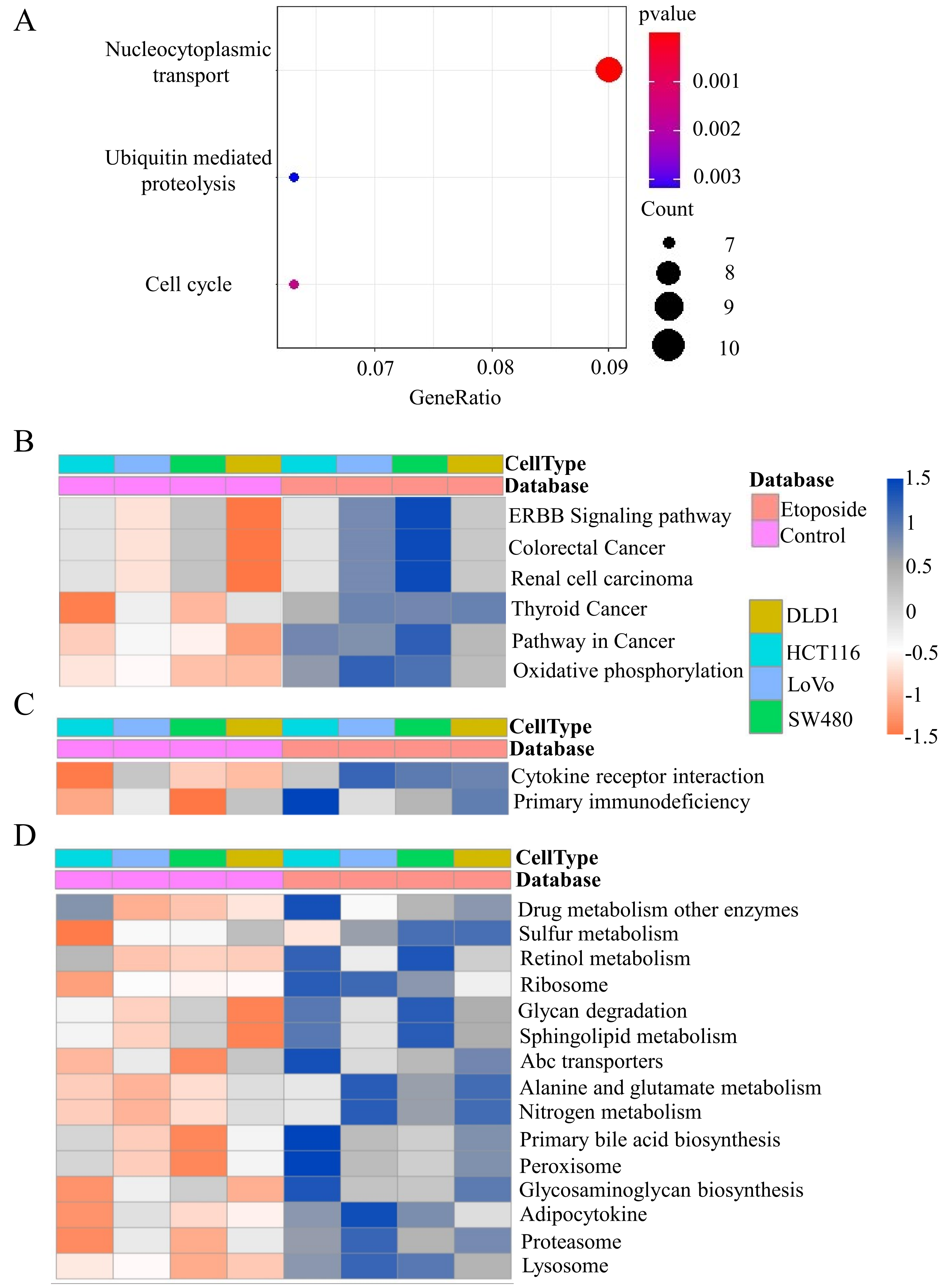

Supplementary Figure 3

A

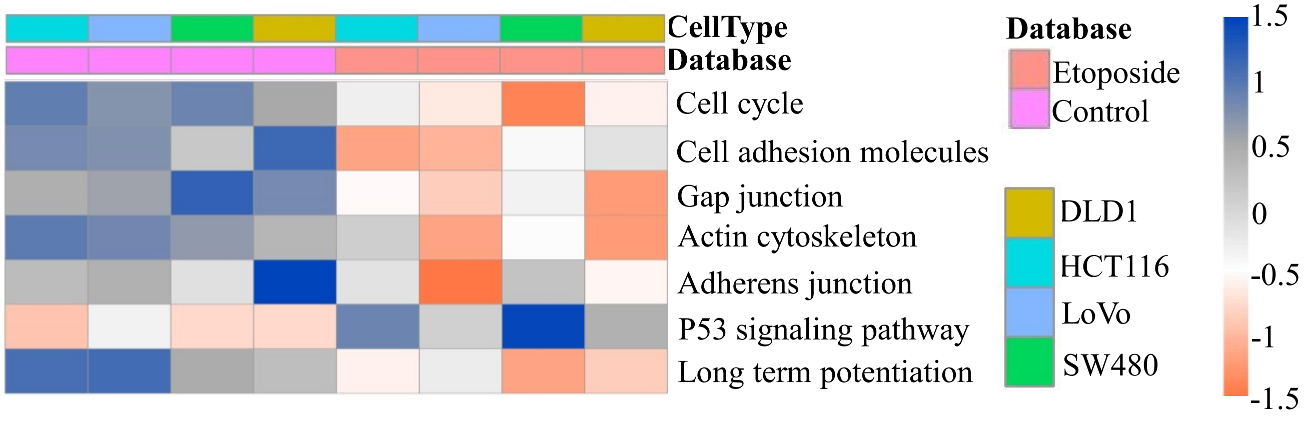

B

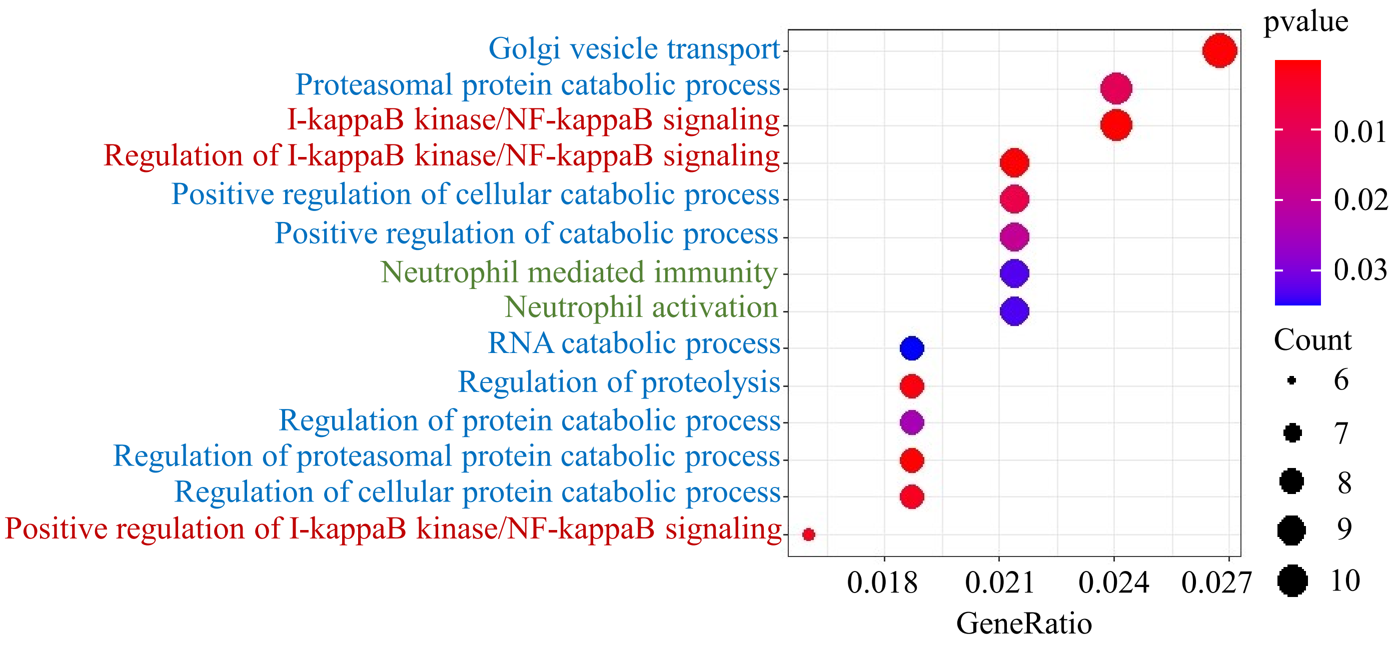

Supplementary Figure 4

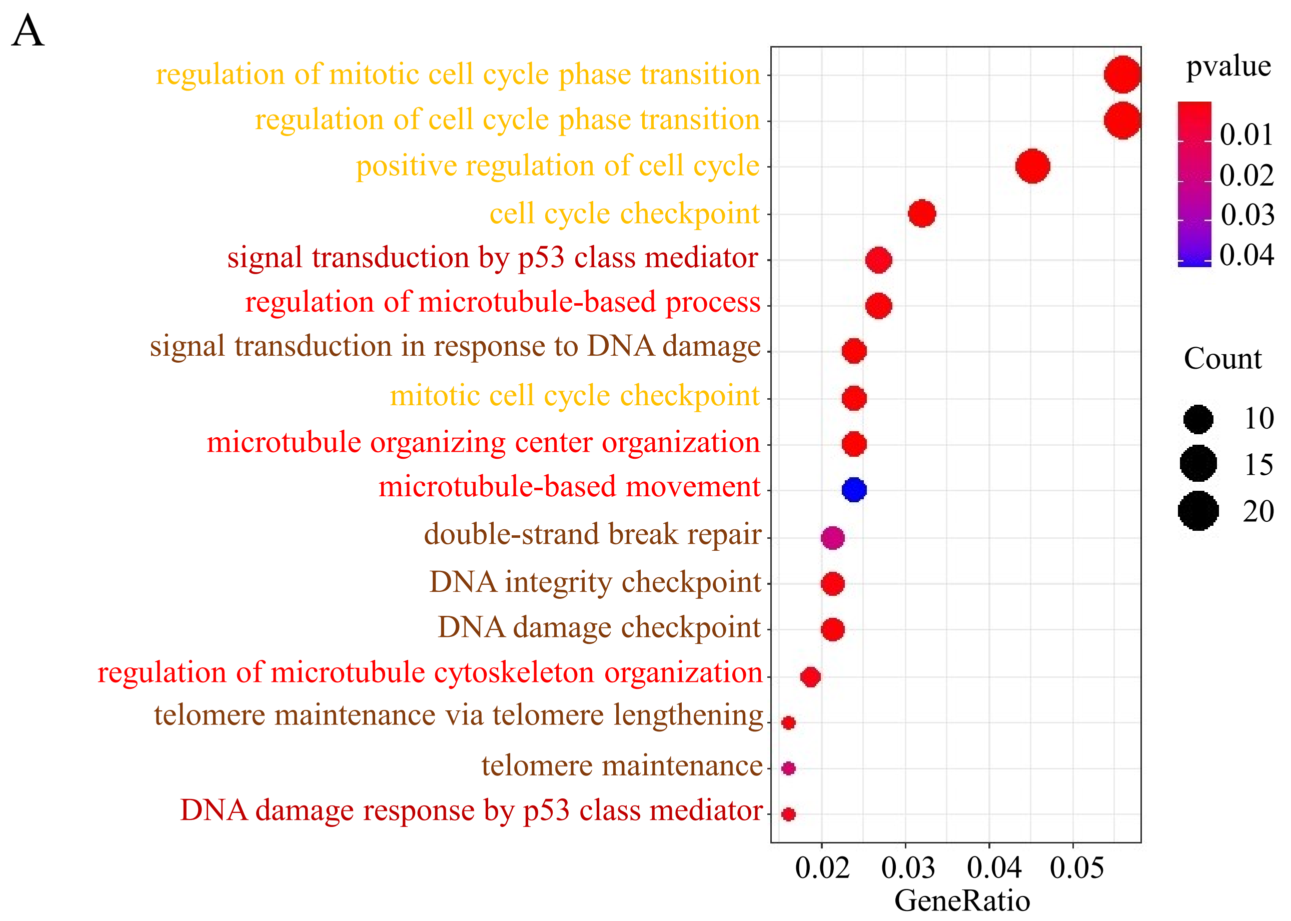

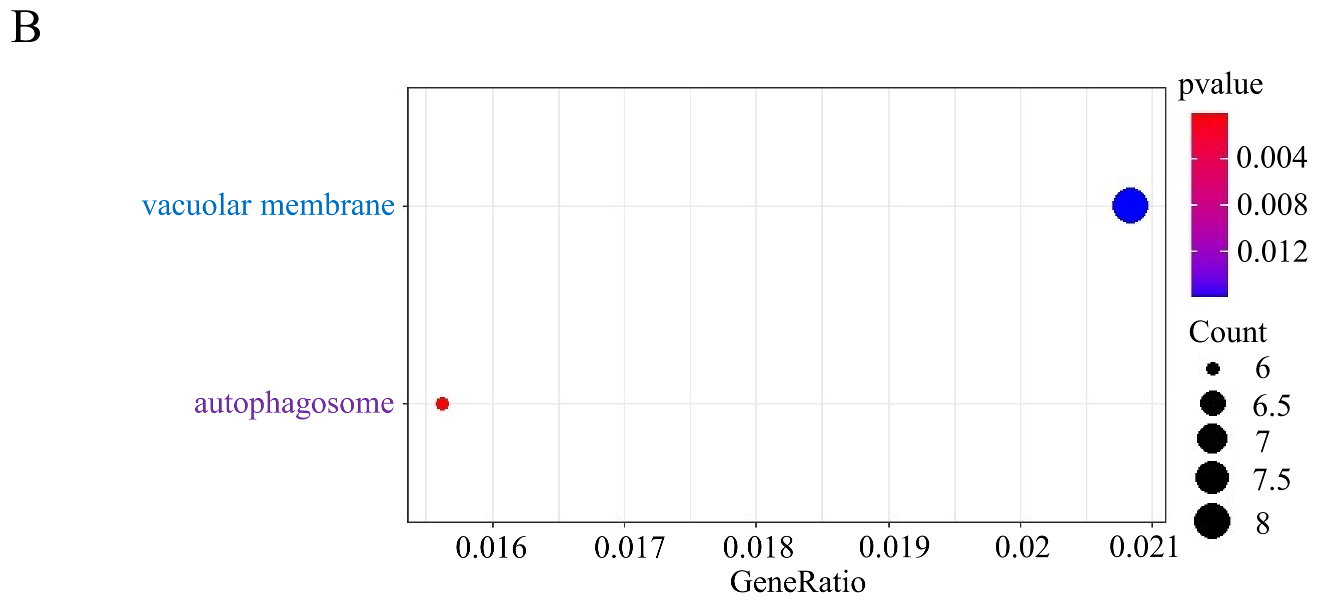

Supplementary Figure 5

A

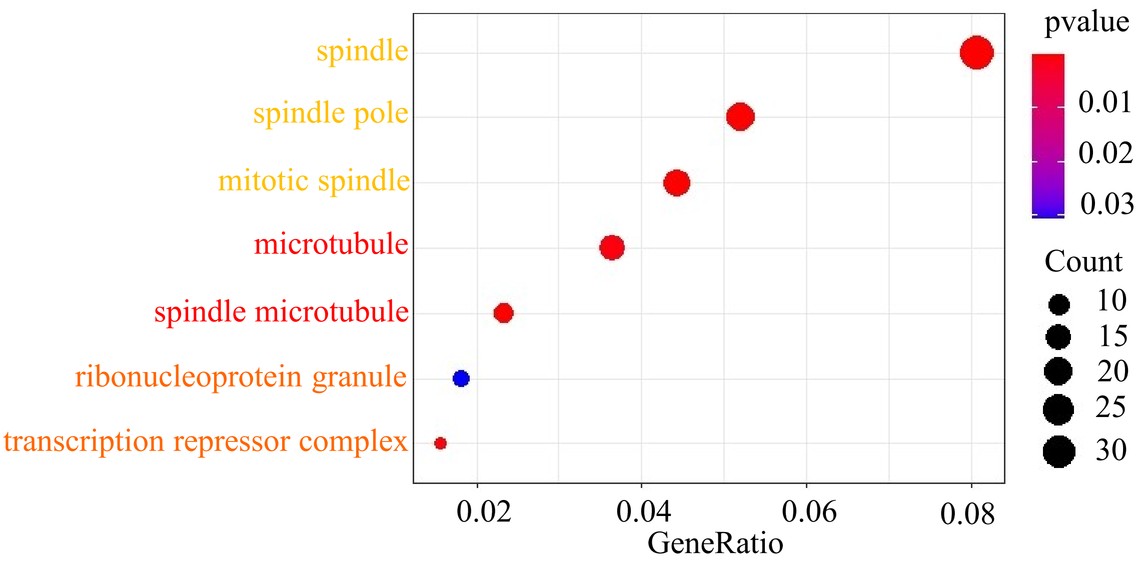

B

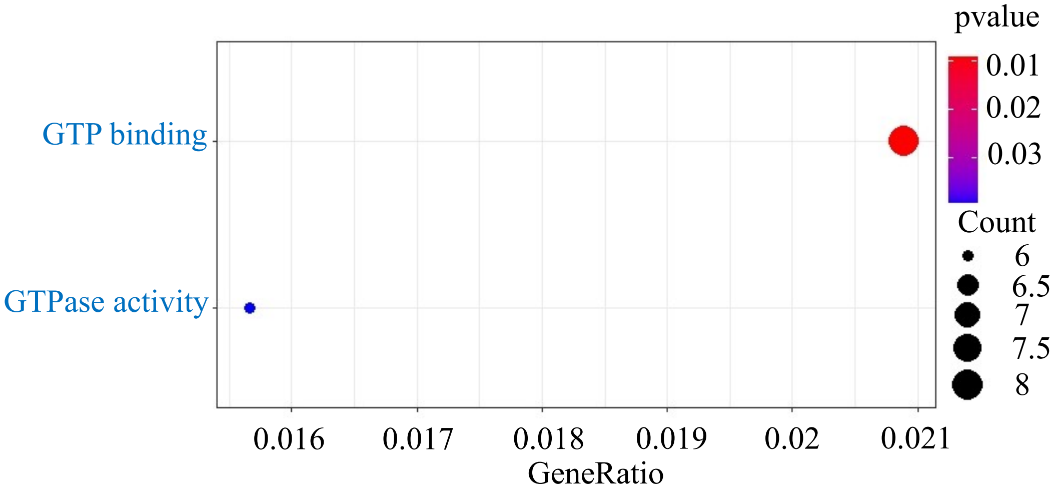

C

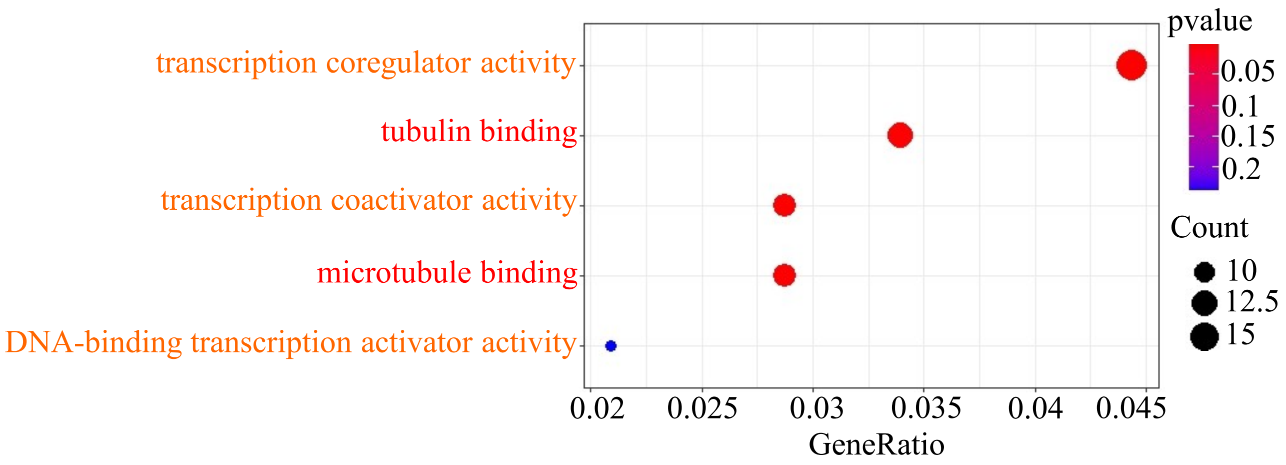

Supplementary Figure 6

A

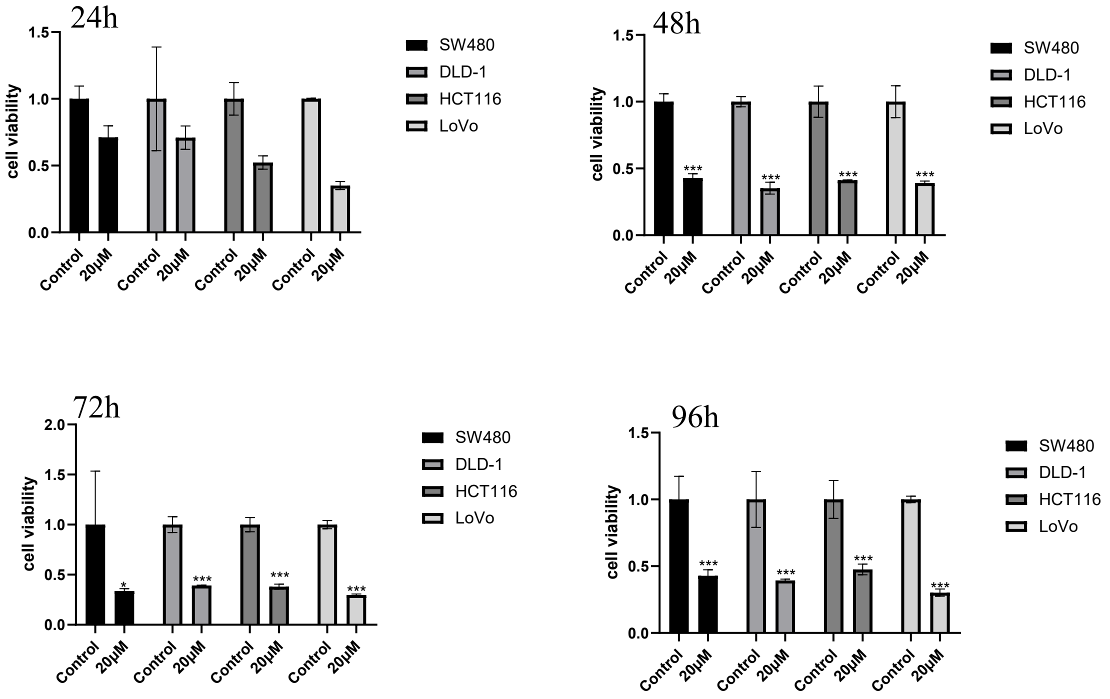

B

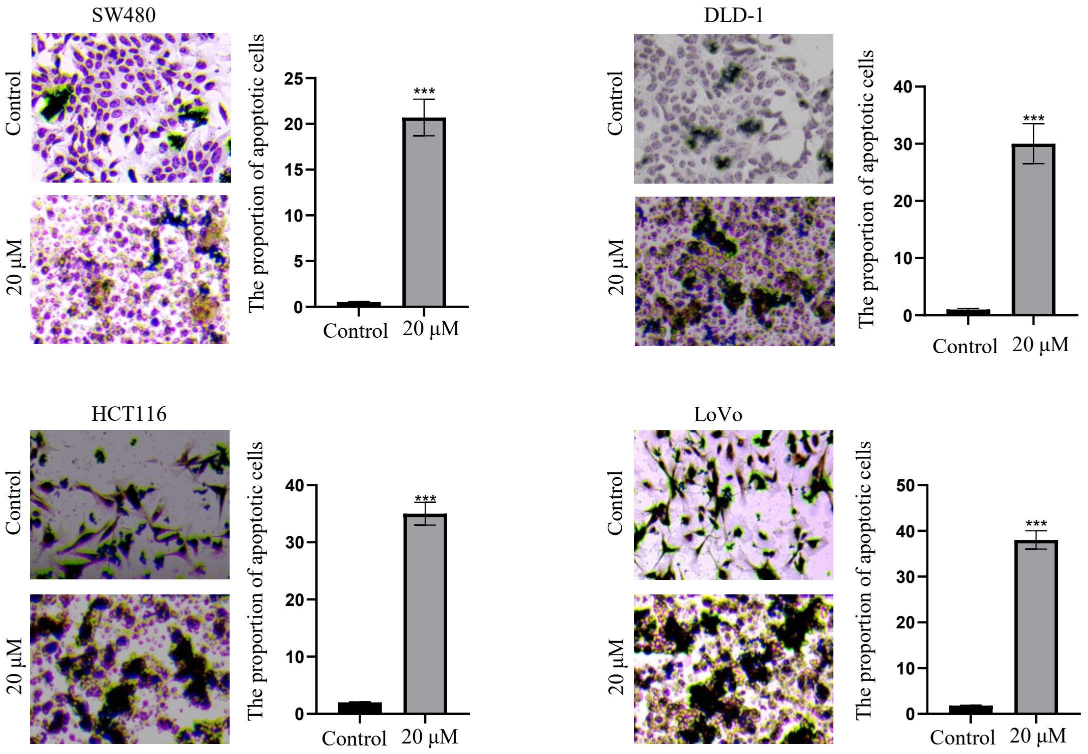

Supplementary Materials

Supplementary Figure 1:

(A): Analysis of significantly enriched DEGs found three downregulated gene sets. Volcano plot of 6924 genes. Green and yellow plots represent genes with fold change ≥ 1.2 or ≤ -1.2, respectively, with $p < 0.05$. Pink plots represent the rest of the genes with no significant expression change. Eight genes identified using PPI analysis are labeled.

Supplementary Figure 2:

(A): Analysis of significantly enriched downregulated DEGs from KEGG analysis in the oncogene signaling pathways, immunity signaling pathways and metabolism-related signaling pathways.

(B): Analysis of significantly enriched upregulated DEGs from KEGG analysis in the oncogene signaling pathways.

(C): Analysis of significantly enriched upregulated DEGs from KEGG analysis in the immunity signaling pathways.

(D): Analysis of significantly enriched upregulated DEGs from KEGG analysis in the metabolism-related signaling pathways.

Supplementary Figure 3:

(A): Analysis of significantly enriched upregulated DEGs from KEGG analysis in the cell-related signaling pathways.

(B): Significantly enriched GO terms obtained from Gene Ontology biological process analysis in the 14 upregulated gene sets.

Supplementary Figure 4:

(A): Significantly enriched GO terms obtained from Gene Ontology biological process analysis in the 17 downregulated gene sets.

(B): Significantly enriched GO terms obtained from Gene Ontology cellular component analysis in the 2 upregulated gene sets.

Supplementary Figure 5:
(A): Significantly enriched GO terms obtained from Gene Ontology cellular component analysis in the 7 downregulated gene sets.
(B): Significantly enriched GO terms obtained from Gene Ontology molecular function analysis in the 2 upregulated gene sets.
(C): Significantly enriched GO terms obtained from Gene Ontology molecular function analysis in the 5 downregulated gene sets.
Supplementary Figure 6:
(A): Proliferation assay in SW480 、 DLD-1 、HCT116 and LoVo cells after etoposide treatment (100×) (***P < 0.001，*P < 0.05).
(B): Apoptosis staining assay in SW480 、 DLD-1 、HCT116 and LoVo cells after etoposide treatment at 12 h (100×)(***P < 0.001).
Supplementary Table 1:
Mutability spectrum of the four cell lines from the Cancer Cell Line Encyclopedia.
Supplementary Table 2:
Data of the 6924 identified differentially expressed genes.
Supplementary Table 3:
Biological Process analysis data of the GO analysis.
Supplementary Table 4:
Cellular Component analysis data of the GO analysis.
Supplementary Table 5:
Molecular Function analysis data of the GO analysis.
